# Favipiravir-Induced Drug Fever in a Young Adult COVID-19 Patient

**DOI:** 10.7759/cureus.14928

**Published:** 2021-05-09

**Authors:** Dhigishaba M Jadeja, Nirmit R Patel

**Affiliations:** 1 Internal Medicine, Gujarat Adani Institute of Medical Sciences, Bhuj, IND; 2 Internal Medicine, Gujarat Cancer Society Medical College, Ahmedabad, IND

**Keywords:** favipiravir, fever, covid-19

## Abstract

Severe acute respiratory syndrome coronavirus 2 has had an indelible effect, with 153,738,171 cases recorded globally as per the World Health Organization’s dashboard. The medical establishment is racing to find repurposed medications that can be successful against this novel coronavirus due to a shortage of new drugs to treat the disease. Favipiravir, an antiviral drug originally developed for influenza, is one of the drugs that has recently received a lot of attention, particularly in India. Here, we present a case of favipiravir-induced drug fever in a young adult coronavirus disease 2019 patient.

## Introduction

As of February 23, 2021, the novel causative virus, the severe acute respiratory syndrome coronavirus 2 (SARS-CoV-2), has affected 13,060,542 people and caused 167,642 deaths, with a case fatality rate of 1.28% in India, which was 1.60% on September 22, 2020 [1]. Coronavirus disease 2019 (COVID-19) has induced a negative effect on social, economic, and psychological well-being in addition to causing physical discomfort [2]. Hypercytokinemia, which usually appears in the second week of COVID-19 and is associated with immunodeficiency as well as hyperinflammation, also known as a cytokine storm, is a feature of SARS-CoV-2 [3]. COVID-19 was previously believed to be a pulmonary illness, but studies have shown that it also affects other tissues in the body [4-6]. Furthermore, several studies have been reported in the literature regarding the symptomatic reinfection or extrapulmonary complication in previously recovered COVID-19 patients [7,8]. Owing to a lack of new medicines to combat the disease, the medical community is racing to identify repurposed antivirals that may be effective against this novel coronavirus. Remdesivir may be a novel antiviral therapy, but its effectiveness is unknown. Favipiravir, an antiviral drug originally developed for influenza, is one of the drugs that has recently received a lot of consideration, particularly in India. However, there is a scarcity of information on adverse reactions and limited sources are available on favipiravir-induced drug fever. Drug fever is difficult to diagnose in such a disease that presents with fever, especially if the drug is a new treatment for an evolving infectious disease like COVID-19. Here, we present a case of favipiravir-induced drug fever in a young adult COVID-19 patient.

## Case presentation

A 27-year-old male presented to the COVID care clinic with the complaint of fever, weakness, backache, headache, and dry cough. The patient’s past medical history was unremarkable.

The patient began experiencing weakness on February 24th, 2021. On the third day of illness, with the emergence of backache, headache, and dry cough, he came to the COVID care center. His vital signs on presentation showed blood pressure of 121/89 mmHg, heart rate of 84 beats per minute, a temperature of 37.8°C, respiratory rate of 18 breaths per minute, and arterial oxygen saturation of 97% (room air). His physical examination was unremarkable, but the patient was lethargic. He tested positive for SARS-CoV-2 by reverse transcriptase-polymerase chain reaction (RT-PCR) from a nasopharyngeal swab. Laboratory data showed normal white blood cell counts, reduced lymphocytes, lactate dehydrogenase (LDH) of 230 U/L (reference range: 135-225 U/L), and C-reactive protein (CRP) of 24 mg/L. His D-dimer level was within the reference range.

**Figure 1:**
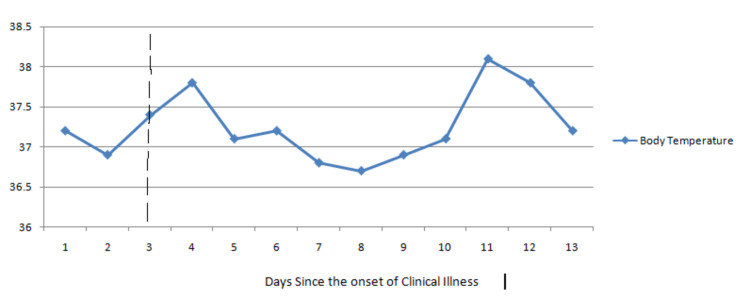
Timeline of favipiravir administration and body temperature. Scattered line represents the clinical illness on the day of favipiravir administration.

On the fourth day of positive RT-PCR for SARS-CoV-2, he was started on favipiravir (1,800 mg twice on the first day followed by 600 mg thereafter), azithromycin 500 mg, and other supportive therapy, including a multivitamin. His clinical condition including fever and other symptoms improved on the sixth day of clinical illness. He remained afebrile until the 10th day of clinical illness. Then, he developed a fever of 38.2°C, thought to be caused by bacterial pneumonia or favipiravir-induced drug fever. Laboratory findings on the 11th day of illness showed normal complete blood count, CRP, LDH, interleukin-6, and procalcitonin. Repeated blood and urinary cultures revealed no evidence of a new infectious source. The chest X-ray was unremarkable. Hence, we diagnosed favipiravir-induced drug fever. The patient’s fever eventually decreased after discontinuing favipiravir, and there was no worsening of symptoms. His fever was relieved without antimicrobial treatment and he was discharged on the following day (Figure 1).

## Discussion

Favipiravir inhibits RNA polymerase, which is responsible for gene replication in RNA virus cells, and thereby prevents viral replication [9]. In vitro, favipiravir has moderate antiviral activity against SARS-CoV-2, an RNA virus, and is predicted to be a COVID-19 therapeutic agent [10]. Elevated blood uric acid levels are a common favipiravir side effect; however, levels rapidly return to normal after the drug is stopped, and most studies found few hyperuricemia symptoms [11]. Cases of drug fever caused by favipiravir, such as the one described here, are also rare. Drug fever is characterized as a fever that occurs with the administration of a drug and disappears after the drug is stopped where no other source can be found after a thorough physical examination and adequate laboratory tests [12]. Patients usually experience fever several days after taking the causative drug, and it goes down within 48-72 hours after stopping the drug [13,14]. In the present case, there was no other possible source of pyrexia observed other than favipiravir. If pyretolysis is not confirmed within 72 hours of stopping the medication, the diagnosis of drug fever may be ruled out [15]. In patients with COVID-19, treated with favipiravir, fever developed after hospital admission on day 11, according to a previous study [16]. In addition, secondary infection is common in COVID-19 patients [12]. Furthermore, as there is an increasing trend of telemedicine during the pandemic [17], it is important to diagnose such drug fever as early as possible when the patient is in self-isolation. These results indicate that determining the therapeutic progress in COVID-19 requires assessing a new-onset fever during therapy. The risk of favipiravir-induced fever should be kept in mind to prevent excessive antiviral or antimicrobial therapy.

## Conclusions

Fever is an adverse effect of favipiravir, and a drug fever should be considered if a fever occurs during favipiravir therapy in COVID-19 patients. This preliminary report could aid in the diagnosis of fever in COVID-19 patients taking favipiravir. Early accurate diagnosis can not only eliminate ineffective, potentially dangerous, and costly diagnostic and therapeutic treatments but may also prevent excessive hospital isolation and bed occupancy, as well as save healthcare workers’ time and effort.
